# Diabetic Cataract—Pathogenesis, Epidemiology and Treatment

**DOI:** 10.1155/2010/608751

**Published:** 2010-06-17

**Authors:** Andreas Pollreisz, Ursula Schmidt-Erfurth

**Affiliations:** Department of Ophthalmology and Optometry, Medical University Vienna, Waehringer Guertel 18-20, 1090 Vienna, Austria

## Abstract

Cataract in diabetic patients is a major cause of blindness in developed and developing countries. The pathogenesis of diabetic cataract development is still not fully understood. Recent basic research studies have emphasized the role of the polyol pathway in the initiation of the disease process. Population-based studies have greatly increased our knowledge concerning the association between diabetes and cataract formation and have defined risk factors for the development of cataract. Diabetic patients also have a higher risk of complications after phacoemulsification cataract surgery compared to nondiabetics. Aldose-reductase inhibitors and antioxidants have been proven beneficial in the prevention or treatment of this sightthreatening condition in in vitro and in vivo experimental studies. This paper provides an overview of the pathogenesis of diabetic cataract, clinical studies investigating the association between diabetes and cataract development, and current treatment of cataract in diabetics.

## 1. Introduction

Worldwide more than 285 million people are affected by diabetes mellitus. This number is expected to increase to 439 million by 2030 according to the International Diabetes Federation. 

A frequent complication of both type 1 and type 2 diabetes is diabetic retinopathy, which is considered the fifth most common cause of legal blindness in the United States [1]. In 95% of type 1 diabetics and 60% of type 2 diabetics with disease duration longer than 20 years, signs of diabetic retinopathy occur. More severe cases of proliferative diabetic retinopathy are seen in patients suffering from type 1 diabetes. Tight control of hyperglycemia, blood lipids, and blood pressure has been shown to be beneficial to prevent its development or progression [2–4].

Cataract is considered a major cause of visual impairment in diabetic patients as the incidence and progression of cataract is elevated in patients with diabetes mellitus [5, 6]. The association between diabetes and cataract formation has been shown in clinical epidemiological and basic research studies. Due to increasing numbers of type 1 and type 2 diabetics worldwide, the incidence of diabetic cataracts steadily rises. Even though cataract surgery, the most common surgical ophthalmic procedure worldwide, is an effective cure, the elucidation of pathomechanisms to delay or prevent the development of cataract in diabetic patients remains a challenge. Furthermore, patients with diabetes mellitus have higher complication rates from cataract surgery [7]. Both diabetes and cataract pose an enormous health and economic burden, particularly in developing countries, where diabetes treatment is insufficient and cataract surgery often inaccessible [8].

## 2. Pathogenesis of Diabetic Cataract

The enzyme aldose reductase (AR) catalyzes the reduction of glucose to sorbitol through the polyol pathway, a process linked to the development of diabetic cataract. Extensive research has focused on the central role of the AR pathway as the initiating factor in diabetic cataract formation. 

It has been shown that the intracellular accumulation of sorbitol leads to osmotic changes resulting in hydropic lens fibers that degenerate and form sugar cataracts [9, 10]. In the lens, sorbitol is produced faster than it is converted to fructose by the enzyme sorbitol dehydrogenase. In addition, the polar character of sorbitol prevents its intracellular removal through diffusion. The increased accumulation of sorbitol creates a hyperosmotic effect that results in an infusion of fluid to countervail the osmotic gradient. Animal studies have shown that the intracellular accumulation of polyols leads to a collapse and liquefaction of lens fibers, which ultimately results in the formation of lens opacities [9, 11]. These findings have led to the “Osmotic Hypothesis” of sugar cataract formation, emphasizing that the intracellular increase of fluid in response to AR-mediated accumulation of polyols results in lens swelling associated with complex biochemical changes ultimately leading to cataract formation [9, 10, 12].

Furthermore, studies have shown that osmotic stress in the lens caused by sorbitol accumulation [13] induces apoptosis in lens epithelial cells (LEC) [14] leading to the development of cataract [15]. Transgenic hyperglycemic mice overexpressing AR and phospholipase D (PLD) genes became susceptible to develop diabetic cataract in contrast to diabetic mice overexpressing PLD alone, an enzyme with key functions in the osmoregulation of the lens [16]. These findings show that impairments in the osmoregulation may render the lens susceptible to even small increases of AR-mediated osmotic stress, potentially leading to progressive cataract formation.

The role of osmotic stress is particularly important for the rapid cataract formation in young patients with type 1 diabetes mellitus [17, 18] due to the extensive swelling of cortical lens fibers [18]. A study performed by Oishi et al. investigated whether AR is linked to the development of adult diabetic cataracts [19]. Levels of AR in red blood cells of patients under 60 years of age with a short duration of diabetes were positively correlated with the prevalence of posterior subcapsular cataracts. A negative correlation has been shown in diabetic patients between the amount of AR in erythrocytes and the density of lens epithelial cells, which are known to be decreased in diabetics compared to nondiabetics suggesting a potential role of AR in this pathomechanism [20].

The polyol pathway has been described as the primary mediator of diabetes-induced oxidative stress in the lens [21]. Osmotic stress caused by the accumulation of sorbitol induces stress in the endoplasmic reticulum (ER), the principal site of protein synthesis, ultimately leading to the generation of free radicals. ER stress may also result from fluctuations of glucose levels initiating an unfolded protein response (UPR) that generates reactive oxygen species (ROS) and causes oxidative stress damage to lens fibers [22]. There are numerous recent publications that describe oxidative stress damage to lens fibers by free radical scavengers in diabetics. However, there is no evidence that these free radicals initiate the process of cataract formation but rather accelerate and aggravate its development. Hydrogen peroxide (H_2_O_2_) is elevated in the aqueous humor of diabetics and induces the generation of hydroxyl radicals (OH–) after entering the lens through processes described as Fenton reactions [23]. The free radical nitric oxide (NO^•^), another factor elevated in the diabetic lens [24] and in the aqueous humor [25], may lead to an increased peroxynitrite formation, which in turn induces cell damage due to its oxidizing properties.

Furthermore, increased glucose levels in the aqueous humor may induce glycation of lens proteins, a process resulting in the generation of superoxide radicals (O_2_^−^) and in the formation of advanced glycation endproducts (AGE) [26]. By interaction of AGE with cell surface receptors such as receptor for advanced glycation endproducts in the epithelium of the lens further O_2_^−^ and H_2_O_2_ are generated [27].

In addition to increased levels of free radicals, diabetic lenses show an impaired antioxidant capacity, increasing their susceptibility to oxidative stress. The loss of antioxidants is exacerbated by glycation and inactivation of lens antioxidant enzymes like superoxide dismutases [28]. Copper-zink superoxide dismutase 1 (SOD1) is the most dominant superoxide dismutase isoenzyme in the lens [29], which is important for the degradation of superoxide radicals (O_2_^−^) into hydrogen peroxide (H_2_O_2_) and oxygen [30]. The importance of SOD1 in the protection against cataract development in the presence of diabetes mellitus has been shown in various in vitro and in vivo animal studies [31–33]. 

In conclusion, a variety of publications support the hypothesis that the initiating mechanism in diabetic cataract formation is the generation of polyols from glucose by AR, which results in increased osmotic stress in the lens fibers leading to their swelling and rupture.

## 3. Clinical Studies Investigating the Incidence of Diabetic Cataract

Several clinical studies have shown that cataract development occurs more frequently and at an earlier age in diabetic compared to nondiabetic patients [34–36].

Data from the Framingham and other eye studies indicate a three to fourfold increased prevalence of cataract in patients with diabetes under the age of 65, and up to a twofold excess prevalence in patients above 65 [34, 37]. The risk is increased in patients with longer duration of diabetes and in those with poor metabolic control. A special type of cataract—known as snowflake cataract—is seen predominantly in young type 1 diabetic patients and tends to progress rapidly. Cataracts may be reversible in young diabetics with improvement in metabolic control. The most frequently seen type of cataract in diabetics is the age-related or senile variety, which tends to occur earlier and progresses more rapidly than in nondiabetics. 

The Wisconsin Epidemiologic Study of Diabetic Retinopathy investigated the incidence of cataract extraction in people with diabetes. Furthermore, additional factors associated with higher risk of cataract surgery were determined. The 10-year cumulative incidence of cataract surgery was 8.3% in patients suffering from type 1 diabetes and 24.9% in those from type 2 diabetes. Predictors of cataract surgery included age, severity of diabetic retinopathy and proteinuria in type 1 diabetics whereas age and use of insulin were associated with increased risk in type 2 diabetics [38].

A follow-up examination of the Beaver Dam Eye Study cohort, consisting of 3684 participants 43 years of age and older, performed 5 years after the baseline evaluation showed an association between diabetes mellitus and cataract formation [39]. In the study, the incidence and progression of cortical and posterior subcapsular cataract was associated with diabetes. In addition, increased levels of glycated hemoglobin were shown to be associated with an increased risk of nuclear and cortical cataracts.

In a further analysis of the Beaver Dam Eye study the prevalence of cataract development was studied in a population of 4926 adults [40]. Diabetic patients were more likely to develop cortical lens opacities and showed a higher rate of previous cataract surgery than nondiabetics. The analysis of the data proved that longer duration of diabetes was associated with an increased frequency of cortical cataract as well as an increased frequency of cataract surgery.

The aim of the population-based cross-sectional Blue Mountains Eye Study was to examine the relationship between nuclear, cortical, and posterior subcapsular cataract in 3654 participants between the years 1992 to 1994 [41]. The study supported the previous findings of the harmful effects of diabetes on the lens. Posterior subcapsular cataract was shown to be statistically significantly associated with diabetes. However, in contrast to the Beaver Dam Eye Study, nuclear cataract showed a weak, not statistically significant, association after adjusting for other known cataract risk factors. 

A population-based cohort study of 2335 people older than 49 years of age conducted in the Blue Mountains region of Australia investigated associations between diabetes and the 5-year incidence of cataract. The results of this longitudinal study conducted by the same group of investigators as the Blue Mountains Eye Study demonstrated a twofold higher 5-year incidence of cortical cataract in participants with impaired fasting glucose. Statistically significant associations were shown between incident posterior subcapsular cataract and the number of newly diagnosed diabetic patients [42].

The Visual Impairment Project evaluated risk factors for the development of cataracts in Australians. The study showed that diabetes mellitus was an independent risk factor for posterior subcapsular cataract when present for more than 5 years [43].

A goal of the Barbados Eye study was to evaluate the relationship between diabetes and lens opacities among 4314 black participants [44]. The authors found that diabetes history (18% prevalence) was related to all lens changes, especially at younger ages.

## 4. Cataract Surgery in Diabetic Patients

Phacomulsification is nowadays the preferred technique in most types of cataract. This technique was developed by Kelman in 1967 and was not widely accepted until 1996 [45]. It results in less postoperative inflammation and astigmatism, more rapid visual rehabilitation and, with modern foldable lenses, a lower incidence of capsulotomy than with the outdated extracapsular surgery. There has been a recent shift in emphasis towards earlier cataract extraction in diabetics. Cataract surgery is advisable before lens opacity precludes detailed fundus examination. 

While the overall outcomes of cataract surgery are excellent, patients with diabetes may have poorer vision outcomes than those without diabetes. Surgery may cause a rapid acceleration of retinopathy, induce rubeosis or lead to macular changes, such as macular edema or cystoid macular edema [46, 47]. The worst outcomes may occur in operated eyes with active proliferative retinopathy and/or preexisting macular edema [48, 49]. 

In diabetics with or without evidence of diabetic retinopathy the blood-aqueous barrier is impaired leading to an increased risk of postoperative inflammation and development of a sight-threatening macular edema, a process that is exacerbated by cataract surgery [50–52]. Factors that influence the amount of postoperative inflammation and the incidence of clinical and angiographic cystoid macular edema are duration of surgery, wound size and posterior capsular rupture or vitreous loss. Liu et al. showed that phacoemulsification surgery affects the blood-aqueous barrier more severely in diabetic patients with proliferative diabetic retinopathy than in patients with nonproliferative diabetic retinopathy or nondiabetic patients [53]. An analysis of Medicare beneficiaries (*n* = 139759) from the years 1997 through 2001 revealed that the rate of cystoid macular edema diagnosis after cataract surgery was statistically significantly higher in diabetic patients than in nondiabetics [54]. 

Several clinical studies investigated the role of phacoemulsification cataract surgery on the progression of diabetic retinopathy. One year after cataract surgery, the progression rate of diabetic retinopathy ranges between 21% and 32% [55–58]. Borrillo et al. reported a progression rate of 25% after a follow-up period of 6 months [59]. A retrospective review of 150 eyes of 119 diabetic patients undergoing phacoemulsification surgery showed a similar progression of diabetic retinopathy in 25% of cases within the follow-up period of 6–10 months [56].

A prospective study evaluating the onset or worsening of macula edema at 6 months following cataract surgery in patients with mild or moderate nonproliferative diabetic retinopathy reported an incidence of 29% (30 of 104 eyes) of macula edema based on angiographic data [60]. Krepler et al. investigated 42 patients undergoing cataract surgery and reported a progression of diabetic retinopathy of 12% in operated versus 10.8% in nonoperated eyes during the follow-up of 12 months [61]. During the same follow-up period of 12 months, Squirrell et al. showed that out of 50 patients with type 2 diabetes undergoing unilateral phacoemulsification surgery 20% of the operated eye and 16% of the nonoperated had a progression of diabetic retinopathy [62]. Liao and Ku found in a retrospective study that out of 19 eyes with preoperative mild to moderate nonproliferative diabetic retinopathy 11 eyes (57.9%) showed progression of diabetic retinopathy 1 year after surgery, while 12 eyes (63.2%) had progressed 3 years postoperatively. The progression rates were statistically significant when compared to eyes without preoperative retinopathy [63]. A recently published prospective study evaluated eyes from 50 diabetic patients with and without retinopathy after cataract surgery by optical coherence tomography [64]. The authors reported an incidence of 22% for macula edema following cataract surgery (11 of 50 eyes) while macula edema did not occur in eyes without retinopathy. When only eyes with confirmed diabetic retinopathy were evaluated (*n* = 26), the incidence for postoperative macula edema and cystoid abnormalities increased to 42% (11 of 26 eyes). Minimal changes from baseline values in center point thickness were observed in eyes with no retinopathy. Eyes with moderate nonproliferative diabetic retinopathy or proliferative diabetic retinopathy developed an increase from baseline of 145 *μ*m and 131 *μ*m at 1 month and 3 month, respectively. The difference in retinal thickening between the 2 groups at 1 and 3 months was statistically significant and among patients with retinopathy inversely correlated with visual acuity improvements.

## 5. Anticataract Treatment

### 5.1. Aldose-Reductase Inhibitors

Aldose reductase inhibitors (ARI) comprise a variety of structurally different compounds like plant extracts, animal tissues or specific small molecules. In diabetic rats, plant flavonids, such as quercitrin or the isoflavone genistein, have delayed diabetic cataract formation [65–68]. Examples of natural products with known AR inhibitory activity are extracts from indigenous plants like Ocimum sanctum, Withania somnifera, Curcuma longa, and Azadirachta indica or the Indian herbal Diabecon [69, 70]. Levels of polyol in the lenses of rats have been reduced by injection of intrinsic ARI containing extracts from human kidney and bovine lenses [71]. Nonsteroidal anti-inflammatory drugs, such as sulindac [72, 73], aspirin [74, 75] or naproxen [76] have been reported to delay cataract in diabetic rats through a weak AR inhibitory activity. 

Several experimental studies support the role of ARI in preventing and not only delaying diabetic cataract formation. In a rat model of diabetes, animals were treated with the AR inhibitor Renirestat [77]. The study reported a reduction of sorbitol accumulation in the lens as compared to untreated diabetic rats. Furthermore, in Ranirestat treated diabetic rats there were no signs of lens damage like degeneration, swelling, or disruption of lens fibers throughout the treatment period in contrast to the untreated group.

In a similar study, diabetic rats were treated with a different ARI, Fidarestat [78]. Fidarestat treatment completely prevented cataractous changes in diabetic animals. In dogs the topically applied ARI Kinostat has been shown to reverse the development of sugar cataracts [79].

Other ARI with a beneficial effect on diabetic cataract prevention encompass Alrestatin [80], Imrestat [81], Ponalrestat [82], Epalrestat [83], Zenarestat [84], Minalrestat [85], or Lidorestat [86].

These studies provide a rationale for a potential future use of ARI in the prevention or treatment of diabetic cataracts.

### 5.2. Antioxidant Treatments of Diabetic Cataracts

As oxidative damage occurs indirectly as a result of polyol accumulation during diabetic cataract formation, the use of antioxidant agents may be beneficial.

A number of different antioxidants have been reported to delay cataract formation in diabetic animals. These include the antioxidant alpha lipoic acid, which has been shown to be effective in both delay and progression of cataract in diabetic rats [87].

Yoshida et al. demonstrated that the combined treatment of diabetic rats with vitamin E, a lipid-soluble and antioxidant vitamin, and insulin synergistically prevented the development and progression of cataracts in the animals [88]. 

Pyruvate, an endogenous antioxidant, has recently gained attention for its inhibitory effect on diabetic cataract formation by reducing sorbitol formation and lipid peroxidation in the lens [89]. A study performed by Varma et al. showed that the incidence of cataract in diabetic rats was lower in the pyruvate-treated group than in the untreated control group [90]. Additionally, the severity of opacities in the pyruvate-treated rats was minor than in the control animals. The beneficial effect of pyruvate in the prevention of cataract is mainly attributed to its effective scavenging ability for reactive oxygen species generated by increased levels of sugars in diabetic animals [91].

However, clinical observations in humans suggest that the effect of antioxidant vitamins on cataract development is small and may not prove to be clinically relevant [92].

### 5.3. Pharmacological Agents for the Treatment of Macular Edema Following Cataract Surgery

Proinflammatory prostaglandins have been shown to be involved in the mechanisms leading to fluid leakage from perifoveal capillaries into the extracellular space of the macular region [93]. Due to the ability of topical nonsteroidal anti-inflammatory drugs (NSAIDs) to block the cyclooxygenase enzymes responsible for prostaglandin production, studies suggested that NSAIDs may also reduce the incidence, duration and severity of cystoid macular edema [94–97] by inhibiting the release and breakdown of the blood-retina barrier [98, 99].

Nepafenac, a topical NSAID indicated for the prevention and treatment of anterior segment pain and inflammation after cataract surgery, has been used recently in clinical trials to test its efficacy in reducing the incidence of macular edema after cataract surgery. The active ingredient is a prodrug that rapidly penetrates the cornea to form the active metabolite, amfenac, by intraocular hydrolases particularly in the retina, ciliary body epithelium and choroid [100]. 

A retrospective study compared the incidence of macular edema after uneventful phacoemulsification between 240 patients treated for 4 weeks with topical prednisolone and 210 patients treated with a combination of prednisolone and nepafenac for the same time. The authors concluded that patients treated with topical prednisolone alone had a statistically significantly higher incidence of macular edema than those treated with additional nepafenac [101].
